# Randomized Controlled Trial of a Brief Versus Extended Internet Intervention for Problem Drinkers

**DOI:** 10.1007/s12529-016-9604-5

**Published:** 2016-10-21

**Authors:** John A. Cunningham, Gillian W. Shorter, Michelle Murphy, Vladyslav Kushnir, Jürgen Rehm, Christian S. Hendershot

**Affiliations:** 1 Centre for Addiction and Mental Health, 33 Russell St., Toronto, ON M5S 2S1 Canada; 2 Australian National University, Canberra, Australia; 3 University of Toronto, Toronto, ON Canada; 4 Ulster University, Coleraine, UK; 5 Inspire, Belfast, UK; 6 Teesside University, Middlesbrough, UK; 7 Technische Universität, Dresden, Germany

**Keywords:** Alcohol, Internet intervention, Randomized controlled trial, RCT, Problem drinking

## Abstract

**Purpose:**

Brief Internet interventions have been shown to reduce alcohol consumption. This trial intended to compare the effects of one such brief intervention to an extended Internet intervention for problem drinkers.

**Method:**

Using online advertising, 490 participants, 18 years or older, were recruited and randomized to receive a brief (CheckYourDrinking.net) versus an extended (AlcoholHelpCentre.net) Internet intervention and were followed up at 6, 12, and 24 months. The per protocol primary analysis assessed difference between condition at the 12-month follow-up.

**Results:**

The follow-up rate at 12 months was 83.3 %. ANCOVAs of the primary (Alcohol Use Disorder Identification Test (AUDIT)-C) and secondary outcome variables (drinks in a typical week, highest number of drinks on one occasion—baseline drinking as covariate) revealed no significant (*p* > 0.05) differences between the interventions. Similarly, combined analyses of the 6-, 12-, and 24-month follow-up revealed no significant differences between interventions at all time points.

**Conclusion:**

The present study does not provide support for the added benefit of an extended Internet intervention for problem drinkers over a brief Internet intervention.

## Introduction

There is a growing body of evidence supporting the efficacy of Internet interventions for problem drinkers. While the majority of this work has been conducted with college student samples [1, 2], there is also research investigating the impact of these interventions among adults from the general population who drink in a hazardous fashion. A meta-analysis by Riper et al. [3] found a small effect (*g* = 0.20) of such Internet interventions in general population settings and noted that the size of this effect was similar to that observed in studies investigating the effect of face-to-face brief interventions for problem drinkers in primary care [3].

The majority of studies have only evaluated the impact of Internet interventions at a 6-month follow-up, making assessments of the longer term impact of such interventions a priority [1, 3]. In addition, given that there are now a range of Internet interventions with demonstrated efficacy, it is also important to establish whether extended interventions (i.e., those that contain multiple modules for the participant to use, possibly over several weeks or more) have a greater impact over that observed in a brief Internet intervention (i.e., in this case, a single session intervention). A pilot randomized controlled trial (RCT) conducted to justify the current trial found some support for the added impact of an extended intervention (AlcoholHelpCenter.net (AHC)) over a brief personalized feedback intervention (CheckYourDrinking.net (CYD)) at a 6-month follow-up [4]. The current manuscript will report on the primary outcome of the full AHC versus CYD trial—to establish whether the AHC has a greater impact on drinking compared to the CYD, 12, and 24 months after receiving the intervention.

## Methods

Full details of the trial protocol are published elsewhere [5]. Briefly, potential participants were recruited by using print and online advertisements. Those interested accessed a link, read a brief description of the trial, and filled in a screening questionnaire and online consent form. Those found eligible and providing consent were postal mailed a thank you letter along with instructions and a password to access the online study intervention portal. Eligibility criterion consisted of being from Canada, 18 years or older, and having an Alcohol Use Disorder Identification Test (AUDIT) score of 8 or more, indicating current hazardous alcohol consumption [6]. Participants who used their individually assigned password to access the study intervention portal were randomized to intervention condition. Access of the study portal was counted as the final recruitment stage of the study. Those who did not access the website were not followed up. Postal mailing of the password was employed in order to promote follow-up rates on the assumption that an offline step in recruitment might select participants more invested in taking part in the trial as compared to an entirely online procedure.

**Fig. 1:**
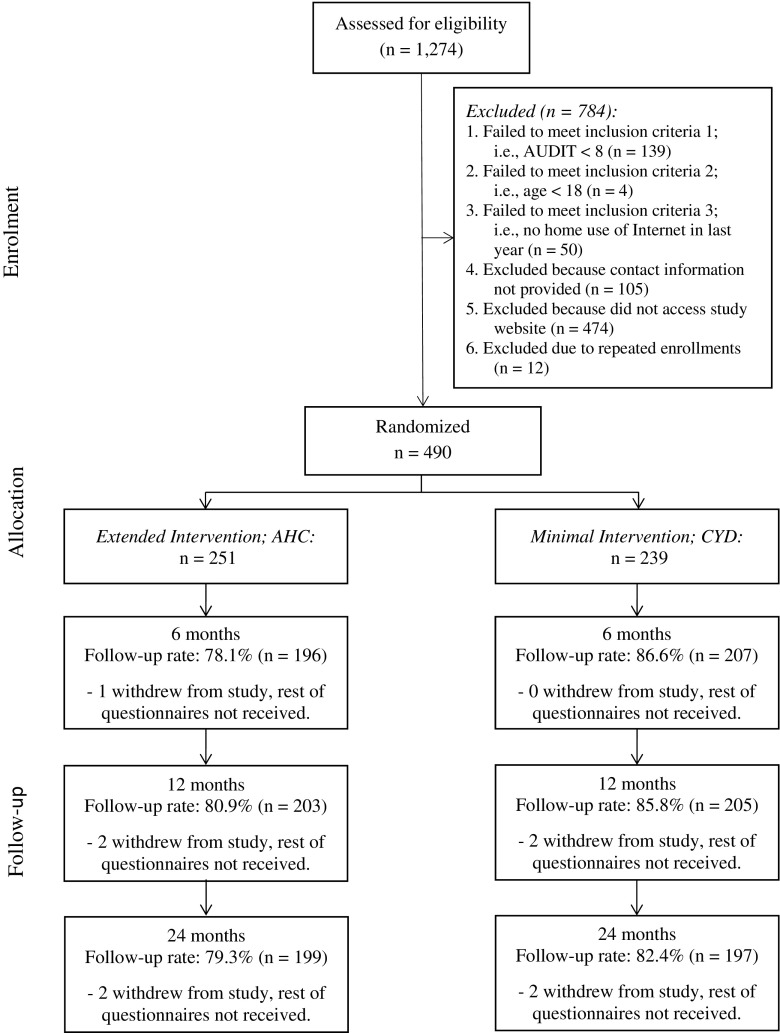
Trial CONSORT flowchart

**Fig. 2:**
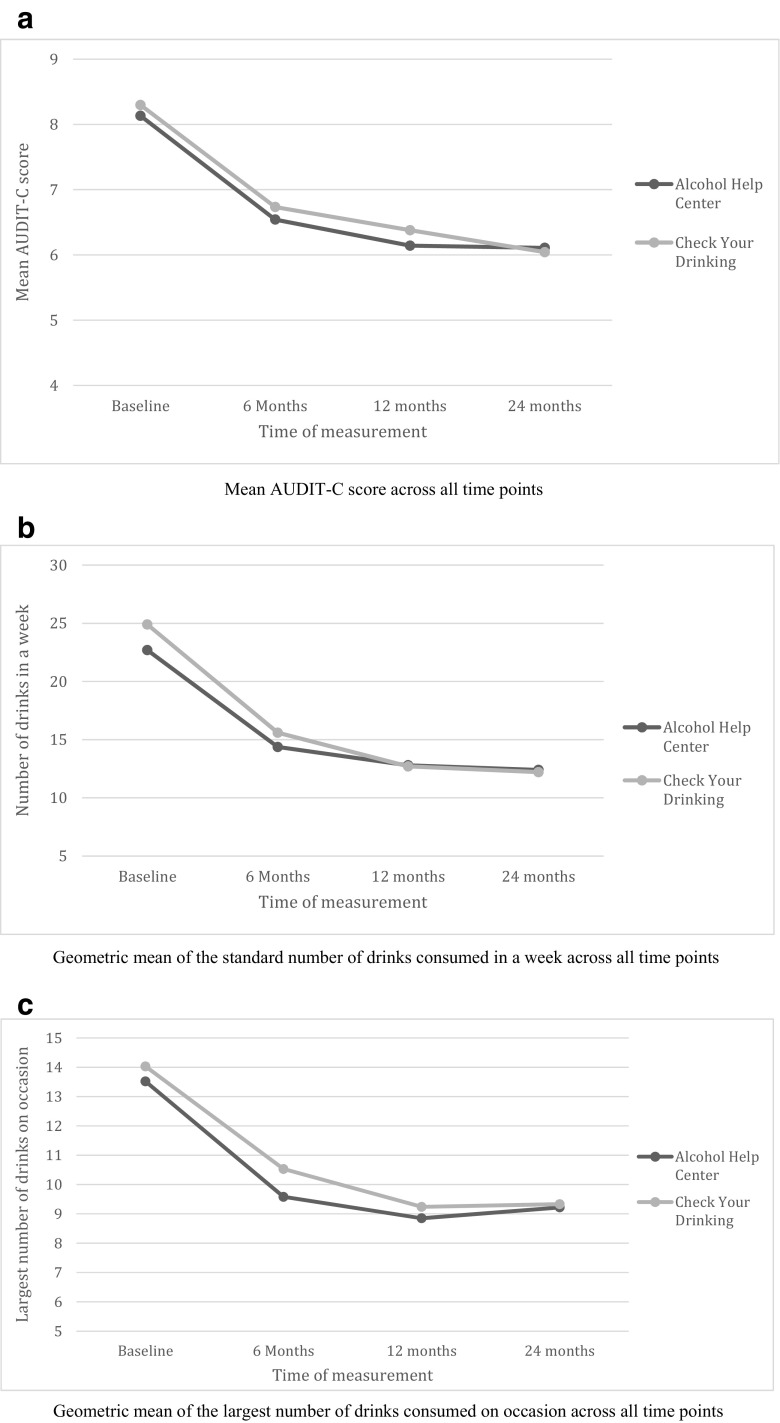
Graphs of means for the three alcohol use variables (imputed data). **a** Mean AUDIT-C score across all time points. **b** Geometric mean of the standard number of drinks consumed in a week across all time points. **c** Geometric mean of the largest number of drinks consumed on occasion across all time points

Follow-ups were conducted at 6, 12, and 24 months (timed based on the participant’s first access of the study intervention portal). Follow-ups were sent by postal mail with those not returning the paper questionnaire within 1 month being sent a link to an online questionnaire via email. Participants were paid Can$20 for the completion of each of the follow-up questionnaires. In addition, in order to reduce loss of potential participants prior to accessing the study intervention portal, those who used their password on the portal were sent a $10 reimbursement. Results from the 12-month follow-up were designated as the primary outcome in the trial protocol and, as such, are analyzed separately from other trial data. See Fig. 1 for a consort chart of the trial (Fig. 2).

### Brief Versus Extended Internet Interventions

Participants were randomly assigned to a brief or extended Internet intervention for hazardous alcohol use. The brief intervention was CYD, a personalized feedback intervention designed to provide feedback on quantity and frequency of drinking and severity of hazardous drinking, that has shown efficacy in reducing alcohol consumption, at least up to a 6-month follow-up, in five RCTs [7–11]. Following the completion of an 18-item screener, the core content of the CYD intervention is personalized normative feedback (providing users with information on how their drinking compares with others in the general population of the same age, sex, and country of origin (for Canada, the USA, and the UK)), summaries of the participants’ drinking, and a report on the severity of current alcohol consumption. The report also includes other useful information on the health effects of alcohol, sensible dinking guidelines, and recommendations for reducing risk of alcohol-related harms.

The extended Internet intervention was the AHC, a multi-session website that contains cognitive behavioral tools modified from treatment and self-help manuals and a moderated support group [4]. The AHC is primarily divided into three sections: (a) getting started (10 exercises focused on initiating change—this section includes a copy of the CYD); (b) dealing with difficulties (6 exercises pertaining to key issues that often occur when working on change, such as dealing with drinking urges and managing relationships); and (c) maintenance (4 exercises designed to help participants maintain their change). In addition, a series of interactive tools are available to help with the change process, such as a drinking diary, where the participant is encouraged to track his or her drinking, a blood alcohol calculator, and e-mail and text messaging educational systems that provide participants with encouragement and tips to deal with drinking concerns. Additional support is also accessible through a support group that is moderated by health educators.

### Outcome Variables

The primary outcome measure for the trial was the AUDIT-C, which comprises the three consumption measures from the AUDIT (frequency, drinks per drinking day, frequency of 5+ drinking days—each asked about the past 6 months) [12, 13]. The AUDIT-C was chosen because it was the primary outcome variable employed in the pilot trial, and because it is a composite measure of drinking variables that are indicative of hazardous alcohol consumption. Secondary outcome measures were number of drinks in a typical week in the past 6 months [14] and highest number of drinks on one occasion in the past 6 months.

### Statistical Analyses

#### Primary Outcome Analysis—12 Months

The per protocol primary outcome analyses were three analysis of covariance (ANCOVA) models. The first was on the primary outcome AUDIT-C at 12 months with experimental condition (CYD or AHC) as between subject variable and baseline value of the outcome variable as the covariate. A similar approach was taken to assess the secondary outcomes—largest number of drinks on an occasion and number of standard drinks consumed in the past week. Analyses were run and presented with missing data excluded and missing data imputed by using maximum likelihood multiple imputation (25 iterations) in SPSS version 22. A list of variables and syntax for the MI procedure can be obtained by contacting the corresponding author. Missing data was characterized as missing at random (MAR, where the missing data may depend on observed data) or missing completely at random (MCAR, where missing data can be considered as random throughout the cases) [15, 16]. Due to non-normality in the secondary outcomes, the data were log transformed (value +1) prior to imputation with the presented estimated marginal means back transformed. The statistician conducting the analysis (GWS) was blind to treatment allocation.

#### Secondary Analyses on Primary and Secondary Outcomes at 0, 6, 12, and 24 Months

As per protocol, the secondary analysis featured AUDIT-C, the number of drinks in a typical week, and the greatest number of drinks on an occasion assessed through a group (AHC, CYD) by time (0, 6, 12, 24 months) interactions in a repeated measures MANOVA design. Missing data was considered MAR or MCAR and treated as above. However, as the data for the secondary outcomes was non-normal, the number of drinks in a typical week and the greatest number of drinks on occasion at all time points were log +1 transformed and back transformed to illustrate geometric means for each of the variables.

### Power Analysis

An a priori power analysis was conducted to determine the sample size needed to detect a one-point difference in AUDIT-C scores (standard deviation of 3) between groups as this was the difference observed in the pilot trial (alpha = 0.05; power = 0.8). The analysis revealed that 143 participants per condition were required for the trial. A 20 % loss to follow-up was anticipated at the 12-month follow-up and a 40 % loss at 24 months; thus, the sample size to be recruited was 480 (based on anticipated 40 % loss to follow-up).

## Results

A total of 490 participants were randomized to condition. The 12-month follow-up rate was 83.3 % with no significant difference between CYD and AHC conditions (*p* > 0.05). Follow-up rates at 6 and 24 months were 82.2 and 80.8 %, respectively. While there was no significant (*p* > 0.05) difference in follow-up rates between condition at 24 months, at 6 months, participants in the brief intervention condition were more likely to provide a follow-up compared to participants in the extended intervention condition (86.6 versus 78.1 %, respectively; Fisher’s exact test, *p* = 0.018). Bivariate comparisons between conditions at baseline also revealed no significant differences in drinking or demographic characteristics *(p* > 0.05—summarized on Table 1). The mean (SD) AUDIT score was 19.5 (7.8), indicating that a substantial proportion of participants may meet criterion for likely alcohol dependence (suggested by a score of 20 or more on the AUDIT).

**Table 1:** Demographic and baseline drinking variables at baseline

	Extended intervention (*n* = 251)	Brief intervention (*n* = 239)	*p*
Mean (SD) age	37.6 (13.9)	37.5 (13.6)	0.94
% Male	50.6	50.2	0.50
% Married/common law	39.8	39.7	0.53
% Some post-secondary education	56.6	59.8	0.26
% Full-time/part-time employed	58.0	58.2	0.52
% Family income Can$<30,000	42.2	40.3	0.37
Mean (SD) AUDIT score	19.4 (7.7)	19.6 (8.0)	0.41
Mean (SD) AUDIT-C score	8.1 (2.0)	8.3 (2.2)	0.12
Mean (SD) largest number of drinks on one occasion^a^	13.5 (1.6)	14.0 (1.6)	0.38
Mean (SD) drinks in a typical week^a^	22.7 (2.0)	24.9 (2.0)	0.15

^a^Bivariate comparison conducted on log transformed (value +1) with means and standard deviations back transformed (geometric means)

### Outcome Analyses

#### Primary Outcome Analyses—12 months

Estimated marginal means for the 12-month follow-up are presented in Table 2. An ANCOVA was conducted for the primary outcome of AUDIT-C score at 12 months controlling for baseline AUDIT-C. There was no significant effect of condition in either imputed data (mean value [range] *F*(1, 487) = 0.38 [0.01–1.61]; *p* = 0.64 [0.21–0.93]; partial *η* ^2^ = 0.000 [0.000–0.003]) or original data (*F*(1, 403) = 0.461; *p* = 0.497; partial *η* ^2^ = 0.001). For secondary outcomes, two separate ANCOVAs were performed on the largest number of drinks on one occasion and on the number of drinks in a week at 12 months while controlling for their corresponding baseline value. There was no difference between conditions for either largest drinks on occasion at 12 months (imputed data mean value [range] *F*(1, 487) = 1.11 [0.001–3.14]; *p* = 0.36 [0.12–0.98]; partial *η* ^2^ = 0.002 [0.000–0.006] or original data *F*(1, 399) = 0.12; *p* = 0.73; partial *η* ^2^ < 0.001) or for the number of drinks per week at 12 months (imputed data mean value [range] *F*(1, 479) = 0.26 [0.09–3.19]; *p* = 0.69 [0.08–0.77]; partial *η* ^2^ = 0.000 [0.000–0.003] or original data *F*(1, 390) = 2.44; *p* = 0.12; partial *η* ^2^ = 0.006).

**Table 2:** Estimated marginal means (SE) for the outcome measures at 12-month follow-up in both imputed and original data for the Alcohol Help Center and Check Your Drinking conditions

	Original data				Imputed data			
	*N*	Mean	95 % confidence interval		*N*	Mean	95 % confidence interval	
Primary outcome AUDIT-C at 12 months								
Alcohol Help Center	202	6.23	5.90	6.56	251	6.24	5.91	6.58
Check Your Drinking	204	6.39	6.06	6.71	239	6.35	6.01	6.68
Secondary outcome largest number of drinks on one occasion at 12 months^a^								
Alcohol Help Center	200	8.95	8.32	9.64	251	9.06	8.38	9.77
Check Your Drinking	202	9.12	8.47	9.82	239	9.18	8.51	9.93
Secondary outcome number of drinks in a typical week at 12 months^b^								
Alcohol Help Center	202	13.71	12.27	15.31	251	13.21	11.83	14.72
Check Your Drinking	204	12.11	10.84	13.52	239	12.30	10.96	13.77

^a,b^Geometric means due to transformation

#### Secondary Analyses on Primary and Secondary Outcomes at 0, 6, 12, and 24 Months

A repeated measures MANOVA model was conducted to test the intervention effect on the three drinking behavior variables (AUDIT-C, highest number of drinks on occasion, and weekly drinking). The results demonstrated no difference between the two intervention groups (AHC and CYD) on drinking behavior over time (original data *F*(9, 2664) = 1.26; *p* = 0.26; partial *η* ^2^ = 0.004; imputed data mean value [range] *F*(9, 4392) = 1.21 [0.69–2.01]; *p* = 0.37 [0.03–0.75]; partial *η* ^2^ = 0.002 [0.001–0.004]. Univariate tests also revealed that there was no intervention effect on AUDIT-C score (original data *F*(2.75, 812.39) = 0.93; *p* = 0.42; partial *η* ^2^ = 0.003; imputed data mean value [range] *F*(2.95, 1437.34) = 0.90 [0.12–2.47]; *p* = 0.52 [0.06–0.95]; partial *η* ^2^ = 0.002 [0.000–0.005]), on the highest number of drinks on occasion (original data *F*(2.79, 813.60) = 1.60; *p* = 0.19; partial *η* ^2^ = 0.005; imputed data mean value [range] *F*(2.88, 1405.15) = 1.07 [0.16–2.98]; *p* = 0.42 [0.03–0.92]; partial *η* ^2^ = 0.002 [0.000–0.006]), and on the number of drinks consumed in a given week (original data *F*(2.76, 816.87) = 2.20; *p* = 0.09; partial *η* ^2^ = 0.007; imputed data mean value [range] *F*(2.92, 1424.91) = 1.21 [0.39–2.89]; *p* = 0.33 [0.04–0.53]; partial *η* ^2^ = 0.003[0.001–0.006]). Note that, due to Box’s and Mauchley’s test violations, the above *F* ratios are the more conservative Pillai’s trace and Huynh-Feldt corrected values [17].

##### Use of the Internet Interventions

Within the first 6 months after baseline, the number of times participants logged into their respective Internet interventions ranged from 1 to 4 times in the CYD condition and 1 to 47 times in the AHC condition. Number of times accessed was categorized to account for the skewed distribution (CYD number of times accessed 1 = 89.1 %, 2 = 9.6 %, 3 = 0.8 %, 4 or more = 0.4 %; AHC number of times accessed 1 = 43 %, 2 = 34.7 %, 3 = 8.8 %, 4 or more = 13.5 %), with participants in the AHC condition accessing the website significantly more often than those in the CYD condition (χ2 = 119.14, 3 df, *p* < 0.001).

## Discussion

There was no evidence that the extended Internet intervention had a greater impact on drinking as compared to the brief intervention. This contrasts with the results of the pilot study used to justify this research where there was a marginally significant advantage of the extended over the brief intervention, albeit at 6 months [4]. Further, while it is true that five previous trials have shown an impact of the CYD brief online intervention [7–11], the current trial did not have a no intervention control condition, making it unwarranted to make the claim that both interventions showed an impact based on these findings.

The lack of any observable difference between brief and extended interventions merits a closer look at the potential limitations of the current study—particularly as the results contrast with those of the pilot trial. The original grant submission included a no intervention control group but this was judged as unnecessary by the review board given the existing evidence base for the CYD intervention. The resulting study’s inability to make conclusions regarding the efficacy of either intervention underlines the risk of the decision to remove a no intervention control group—particularly in a relatively new field, such as Internet intervention research—where it may be inappropriate to be confident in the efficacy of most existing interventions. Other trials addressing similar topics have recognized this and incorporated a no intervention control group in their design [18]. The contrast in the findings between the pilot study and the current one underlines this same statement but also leads to speculations as to what, besides the length of the follow-up and the probability of outcomes associated with a *p* < 0.05 analysis approach, might have led to differences in outcomes. Of particular importance may be that both studies employed convenience samples. The pilot study sample was recruited by paper newspaper advertisements and the current study primarily through online advertisements at different websites. While both samples reported relatively severe levels of alcohol use, other observed demographic characteristics (as well, no doubt, as other unmeasured factors) differed between the samples. It is also possible that the nature of recruitment in the current (and pilot) trials resulted in a sample who were already motivated to change, making the nature and length of the intervention less important to the outcome of the participants. In addition, the handling of missing data was more sophisticated in the current study leading to more confidence in the results. However, one limitation of the current analysis is that, from simulation studies on the non-normal, count variable of weekly drinking, it is noted that the use of SPSS MI may result in lower imputed means than the true value for the population [19]; however, there were similar levels of missing data in each arm at 12-month follow-up. Finally, the severity of the alcohol consumption by participants in the current study may also be a limitation, as people with alcohol dependence could benefit from more formalized help to reduce their consumption. However, in counter-argument to this, the reality of Internet intervention use is that severity of problems does not generally act as an exclusion criterion for public access websites. Thus, it makes sense to evaluate the efficacy of the interventions without restricting the sample based on severity of alcohol use.

There was a significant difference in the amount participants used the respective interventions. The large majority of participants in the CYD condition accessed it once, while participants in the AHC condition were more likely to have returned two or three times (and the minority many more). For participants in the CYD condition, this limited amount of use is understandable, given that the CYD is largely intended as a single access intervention. However, while there was more use of the AHC than the CYD, this is still a very limited utilization of what is a fairly extensive set of cognitive behavioral and relapse prevention tools. Perhaps this lack of use of the AHC suggests that brief Internet interventions are a better match for an online format. Or, the findings could emphasize the need to find ways to promote engagement (such as using reminder emails) and ease of access of online interventions (e.g., online intervention architecture designed to guide the participant), particularly as limited use of any online intervention seems to be the rule rather than the exception [20].

It is important to recognize that this one study does not provide sufficient evidence that the potential efficacy of extended online interventions for problem drinking does not differ from that of brief interventions. There are certainly trials that demonstrate the impact of such extended interventions over a no intervention control [2, 3] but insufficient research exploring brief versus extended Internet interventions. However, it might be possible to consider this trial within the context of other research where the comparator to the extended Internet intervention is a static information control group. The Down Your Drink trial is one example of such a design and, further, one where there were no significant differences observed between conditions [21]. Thus, if the static online control could be regarded as a sort of brief intervention, then there is a larger body of research calling into question whether extended online interventions have any additional impact over brief interventions on participant drinking. A more detailed examination may also be merited of the control groups employed in the extant Internet intervention literature for hazardous alcohol consumption as the nature of the comparator—a brief interactive intervention (such as the CYD employed in the current trial), a static comparison website (such as that used in the Down Your Drink trial), or other types of information only controls summarized in the Riper et al. review [3]—may lead to differences in the statistical significance of any observed difference between groups.

A troubling aspect of concluding that extended online interventions for problem drinking have no increased influence on drinking relative to brief interventions is that some participants do engage with the intervention extensively, and over a long time. Further, some of these participants state that they benefit from this extended use (although there is no unambiguous outcome evidence employing RCTs varying allowable length of use to support this claim). If an extended intervention is already constructed, or not significantly more expensive to construct and maintain than a brief intervention, is it not worth having the intervention available for the participants who, although few in number, might benefit from extended use? In addition, if there was a way to identify who these extended users might be prior to the participants taking part in the trial, is there worth in conducting an RCT to compare the benefits of a brief versus extended intervention among this subgroup? If both types of interventions are available, are there means to allow participants to choose whether they want the short or long version (and options for continuing on to the longer version if the participant decides they want more)? There may also be worth in considering the benefits of “front loading” essential components of extended interventions so that they will be utilized by the largest number of problem drinkers as possible, whether they continue on to the rest of the intervention or not. This same argument has been made with regard to face-to-face treatment, where the majority of participants showing up for treatment do not return for the full treatment program after the first few sessions [22, 23]. The additional advantage of providing essential components of an intervention up front, rather than the expectation that they will be found when people access all sessions, is that providing useful content early on may, in fact, motivate participants to continue on with the treatment, or online intervention, because they are finding it worthwhile [22].

## References

[1] Dedert EA, McDuffie JR, Stein R (2015). Electronic interventions for alcohol misuse and alcohol use disorders a systematic review. Ann Intern Med.

[2] Donoghue K, Patton R, Phillips T, Deluca P, Drummond C (2014). The effectiveness of electronic screening and brief intervention for reducing levels of alcohol consumption: a systematic review and meta-analysis. Journal of medical Internet research..

[3] Riper H, Blankers M, Hadiwijaya H (2014). Effectiveness of guided and unguided low-intensity internet interventions for adult alcohol misuse: a meta-analysis. PLoS One.

[4] Cunningham JA (2012). Comparison of two internet-based interventions for problem drinkers: randomized controlled trial. Journal of medical Internet research..

[5] Cunningham JA, Hendershot CS, Rehm J (2015). Randomized controlled trial of a minimal versus extended internet-based intervention for problem drinkers: study protocol. BMC Public Health.

[6] Saunders JB, Aasland OG, Babor TF, De La Fuente JR, Grant M (1993). Development of the alcohol use disorders identification test (AUDIT): WHO collaborative project on early detection of persons with harmful alcohol consumption— II. Addiction.

[7] Cunningham JA, Wild TC, Cordingley J, van Mierlo T, Humphreys K (2009). A randomized controlled trial of an internet-based intervention for alcohol abusers. Addiction.

[8] Doumas DM, Hannah E (2008). Preventing high-risk drinking in youth in the workplace: a web-based normative feedback program. J Subst Abus Treat.

[9] Doumas DM, Haustveit T (2008). Reducing heavy drinking in intercollegiate athletes: evaluation of a web-based personalized feedback program. The Sport Psychologist.

[10] Doumas DM, McKinley LL, Book P (2009). Evaluation of two web-based alcohol interventions for mandated college students. J Subst Abus Treat.

[11] Cunningham JA, Murphy M, Hendershot CS (2014). Treatment dismantling pilot study to identify the active ingredients in personalized feedback interventions for hazardous alcohol use: randomized controlled trial. Addiction Science and Clinical Practice.

[12] Bush K, Kivlahan DR, McDonell MB, Fihn SD, Bradley KA (1998). The AUDIT alcohol consumption questions (AUDIT-C): an effective brief screening test for problem drinking. Ambulatory care quality improvement project (ACQUIP). Alcohol use disorders identification test. Arch Intern Med.

[13] Dawson DA, Grant BF, Stinson FS, Zhou Y (2005). Effectiveness of the derived alcohol use disorders identification test (AUDIT-C) in screening for alcohol use disorders and risk drinking in the US general population. Alcohol Clin Exp Res.

[14] Romelsjö A, Leifman H, Nyström S (1995). A comparative study of two methods for the measurement of alcohol consumption in the general population. Int J Epidemiol.

[15] Graham JW (2009). Missing data analysis: making it work in the real world. Annu Rev Psychol.

[16] Dziura JD, Post LA, Zhao Q, Fu Z, Peduzzi P (2013). Strategies for dealing with missing data in clinical trials: from design to analysis. The Yale journal of biology and medicine.

[17] Pituch KA, Stevens JP (2016). Applied multivariate statistics for the social sciences: analyses with SAS and IBM’s SPSS.

[18] Sinadinovic K, Wennberg P, Johansson M, Berman AH (2014). Targeting individuals with problematic alcohol use via web-based cognitive-behavioral self-help modules, personalized screening feedback or assessment only: a randomized controlled trial. Eur Addict Res.

[19] Blankers M, Koeter MW, Schippers GM (2010). Missing data approaches in eHealth research: simulation study and a tutorial for nonmathematically inclined researchers. Journal of medical Internet research.

[20] Reinwand DA, Schulz DN, Crutzen R, Kremers SPJ, de Vries H (2015). Who follows eHealth interventions as recommended? A study of Participants’ personal characteristics from the experimental arm of a randomized controlled trial. Journal of medical Internet research.

[21] Wallace P, Murray E, McCambridge J (2011). On-line randomized controlled trial of an internet based psychologically enhanced intervention for people with hazardous alcohol consumption. PLoS One.

[22] Cunningham JA, Sdao-Jarvie K, Koski-Jannes A, Breslin FC (2001). Using self-help materials to motivate change at assessment for alcohol treatment. J Subst Abus Treat.

[23] Pulford J, Adams P, Sheridan J (2006). Unilateral treatment exit: a failure of retention or a failure of treatment fit?. Subst Use Misuse.

